# Supplementary material for: Capsular polysaccharide switching in Streptococcus suis modulates host cell interactions and virulence
Source: Sci Rep. 2021 Mar 22;11:6513. doi: 10.1038/s41598-021-85882-3 (PMC7985379; doi:10.1038/s41598-021-85882-3)
Supplement: Supplementary file 1 — Supplementary Figures. [file 41598_2021_85882_MOESM1_ESM.pdf]

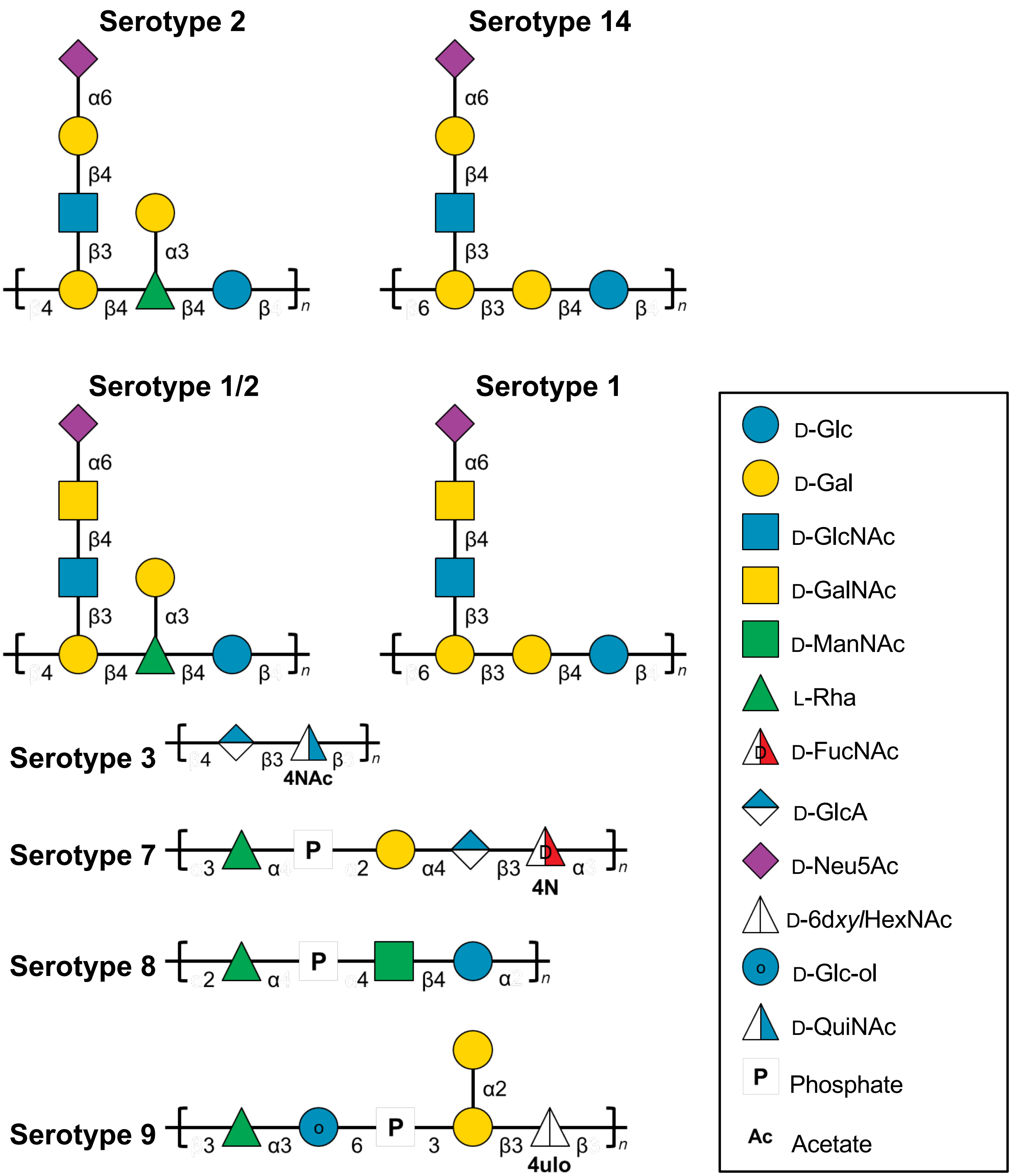

Fig S1. Reported composition and structure of the *S. suis* serotype 1, 2, 1/2, 3, 7, 8, 9, and 14 CPSs. Monosaccharide symbols follow the Symbol Nomenclature for Glycans System (Varki A, Cummings RD, Aebi M, Packer NH, Seeberger PH, Esko JD, et al. Symbol nomenclature for graphical representations of glycans Glycobiology. 2015;25: 1323-1324). The glycosidic linkage positions of carbon of the sugars were shown. Abbreviations: D-6d*xyl*HexNAc, 2-acetamido-2,6-dideoxy-D-*xylo*-hexose; 4NAc, 4-acetamido; 4N, 4-amino; 4ulo, 4-ulo.

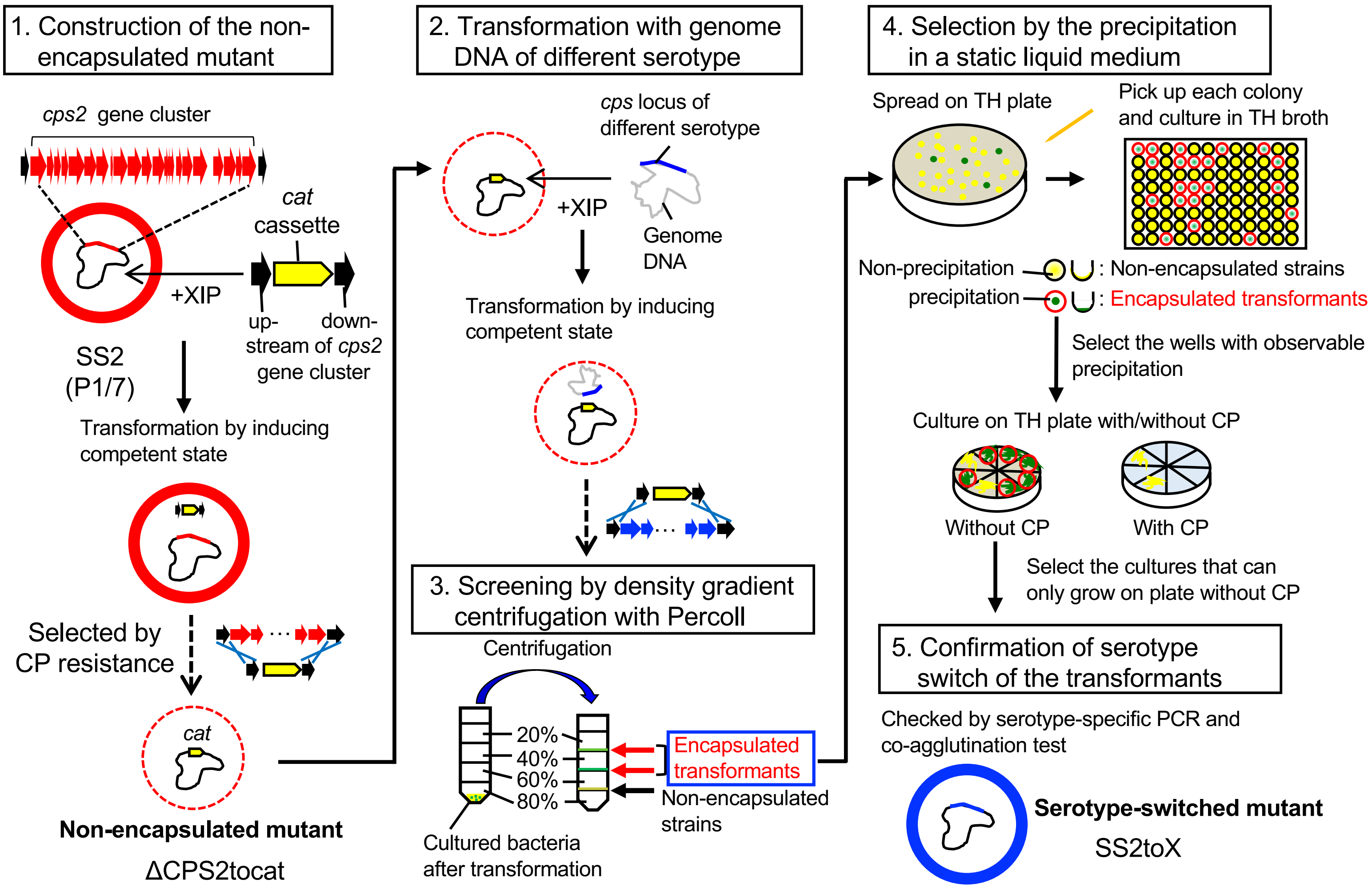

Fig S2. Diagram of the procedure used for construction of the *S. suis* serotype-switched mutants. The procedure consists of five steps. Construction of the non-encapsulated mutant (step ***1***) is the most important step of the procedure, as it is essential for the following screening and selection steps. Due to a lower buoyancy density of encapsulated bacterial cells than those of the non-encapsulated cells, a density gradient centrifugation with Percoll (step ***3***) was used to screen encapsulated (i.e, serotype-switched) transformants from ΔCPS2tocat transformed with genome DNA of a donor strain in step ***2***. Moreover, encapsulated transformants were further selected by the differences in how the precipitations were formed in a static liquid medium (step ***4***) due to the elevated hydrophobicity of non-encapsulated cells. Abbreviation: CP, chroramphenicol; XIP, *sigX*-inducing peptide; TH, Todd-Hewitt.

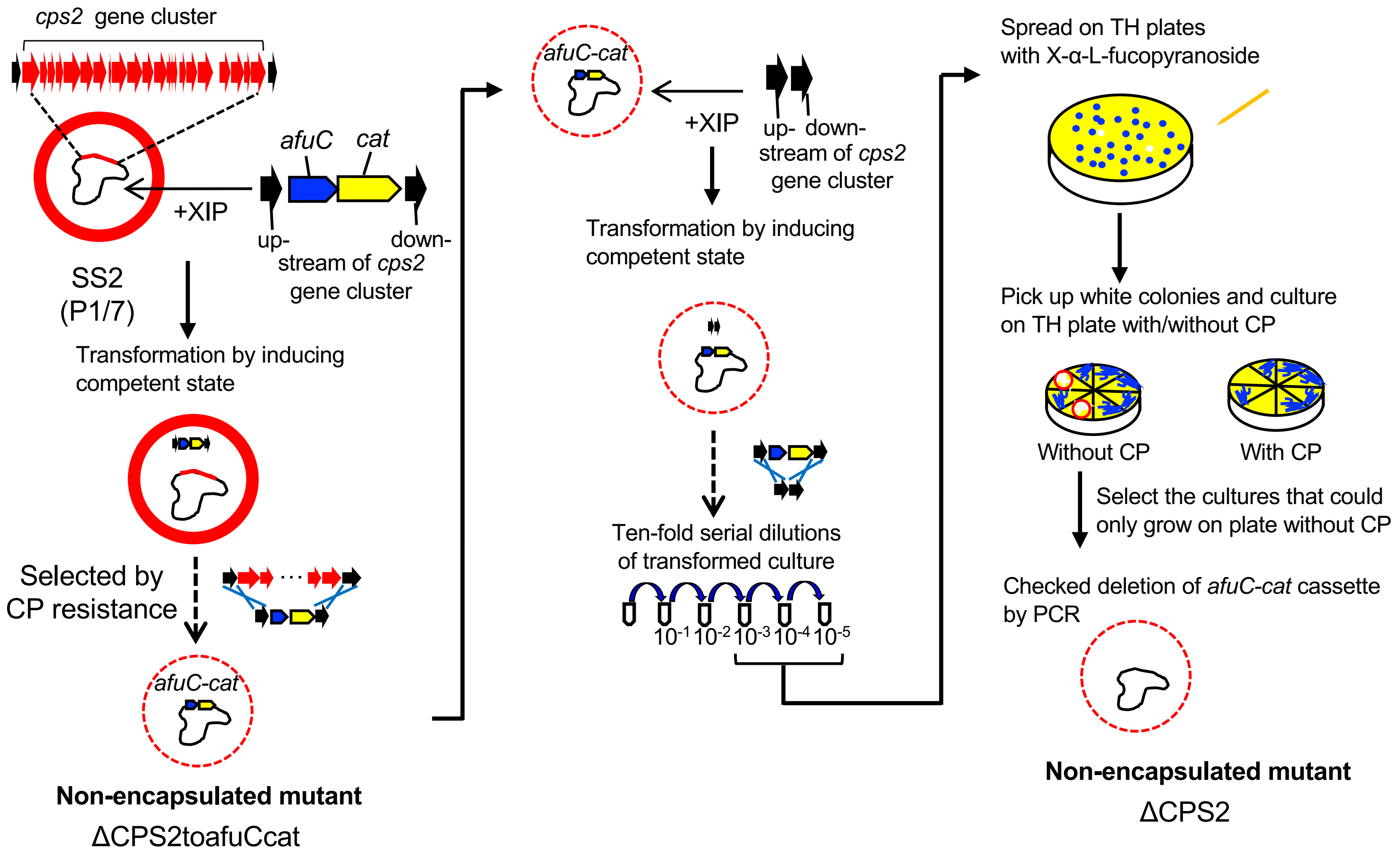

Fig S3. Diagram of the procedure used for construction of the markerless *S. suis* non-encapsulated mutant. Abbreviations: CP, chroramphenicol; XIP, *sigX*-inducing peptide; TH, Todd-Hewitt.

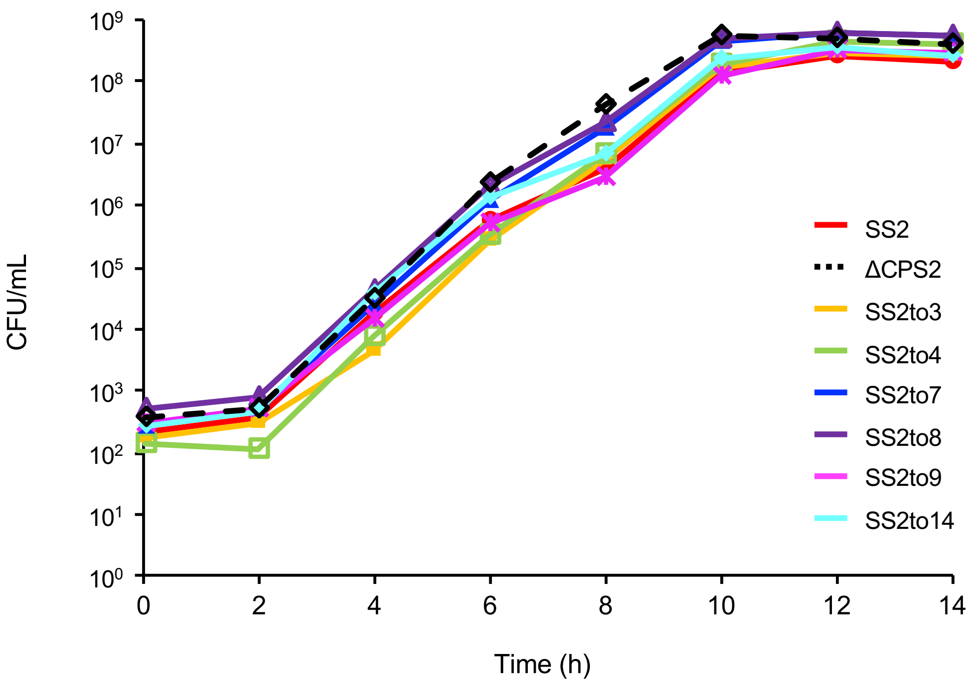

Fig S4. Growth curves of the different *S. suis* serotype-switched mutants. Growth curves of P1/7, non-encapsulated mutant (ΔCPS2) and serotype-switched mutants (SS2to3, SS2to4, SS2to7, SS2to8, SS2to9, and SS2to14) derived from P1/7 are shown.

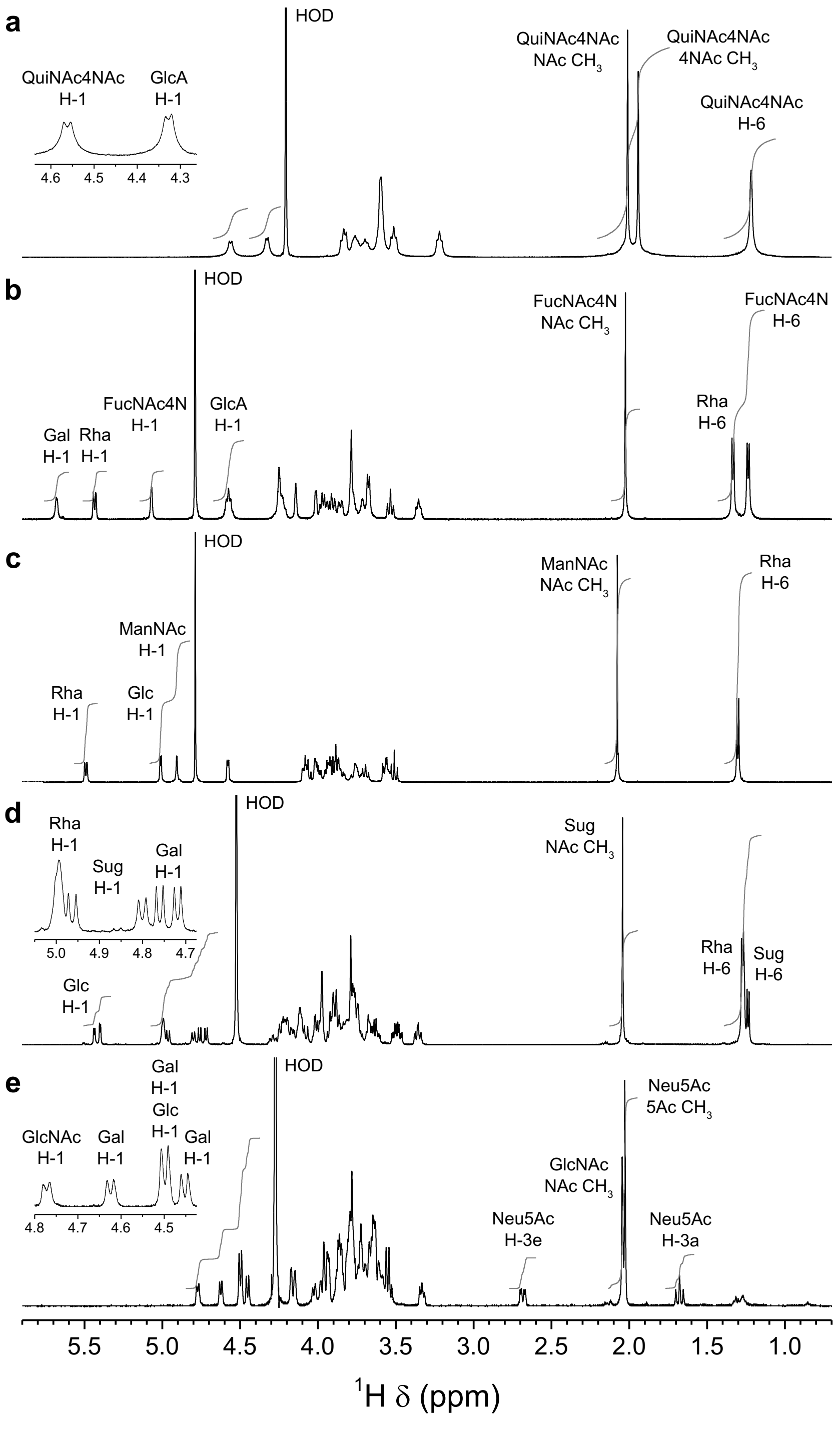

Fig S5. 500 MHz ^{1}H NMR spectra of the *S. suis* serotype-switched mutant CPSs. (**a**) SS2to3, resonance reporter signals (85° C): δ 4.56 and 4.33 (anomeric), 2.01 and 1.94 (acetyl methyl), and 1.22 (6-deoxy sugar methyl); (**b**) SS2to7, resonance reporter signals (25° C): δ 5.68, 5.43, 5.07, and 4.58 (anomeric), 2.02 (acetyl methyl), as well as 1.33 and 1.24 (6-deoxy sugar methyl); (**c**) SS2to8, resonance reporter signals (25° C): δ 5.49, 5.01, and 4.91 (anomeric), 2.08 (acetyl methyl), and 1.30 (6-deoxy sugar methyl); (**d**) SS2to9, resonance reporter signals (50° C): δ 5.44, 5.40, 5.00, 4.99, 4.96, 4.80, 4.76, and 4.72 (anomeric), 2.04 (acetyl methyl), as well as 1.27, 1.27, and 1.24 (6-deoxy sugar methyl); (**e**) SS2to14, resonance reporter signals (77° C): δ 4.77, 4.62, 4.50, 4.50, and 4.45 (anomeric), 2.05 and 2.03 (acetyl methyl), 2.68 (Neu5Ac H-3e), and 1.68 (Neu5Ac H-3a). Except for SS2to9 CPS, the slight differences in chemical shifts compared to published values [references 9, 12, and 13 in the text] can be attributed to different sample concentration and pH, internal reference, and spectral acquisition temperature. Two-dimensional (2D) correlation spectroscopy (COSY) experiments, and additionally for SS2to9 CPS the 2D heteronuclear single-quantum coherence (HSQC) experiment, were also performed, and the observed cross-peaks were in complete support of the structures. Abbreviation: Sug, 6d*xyl*HexNAc-4-ulo.

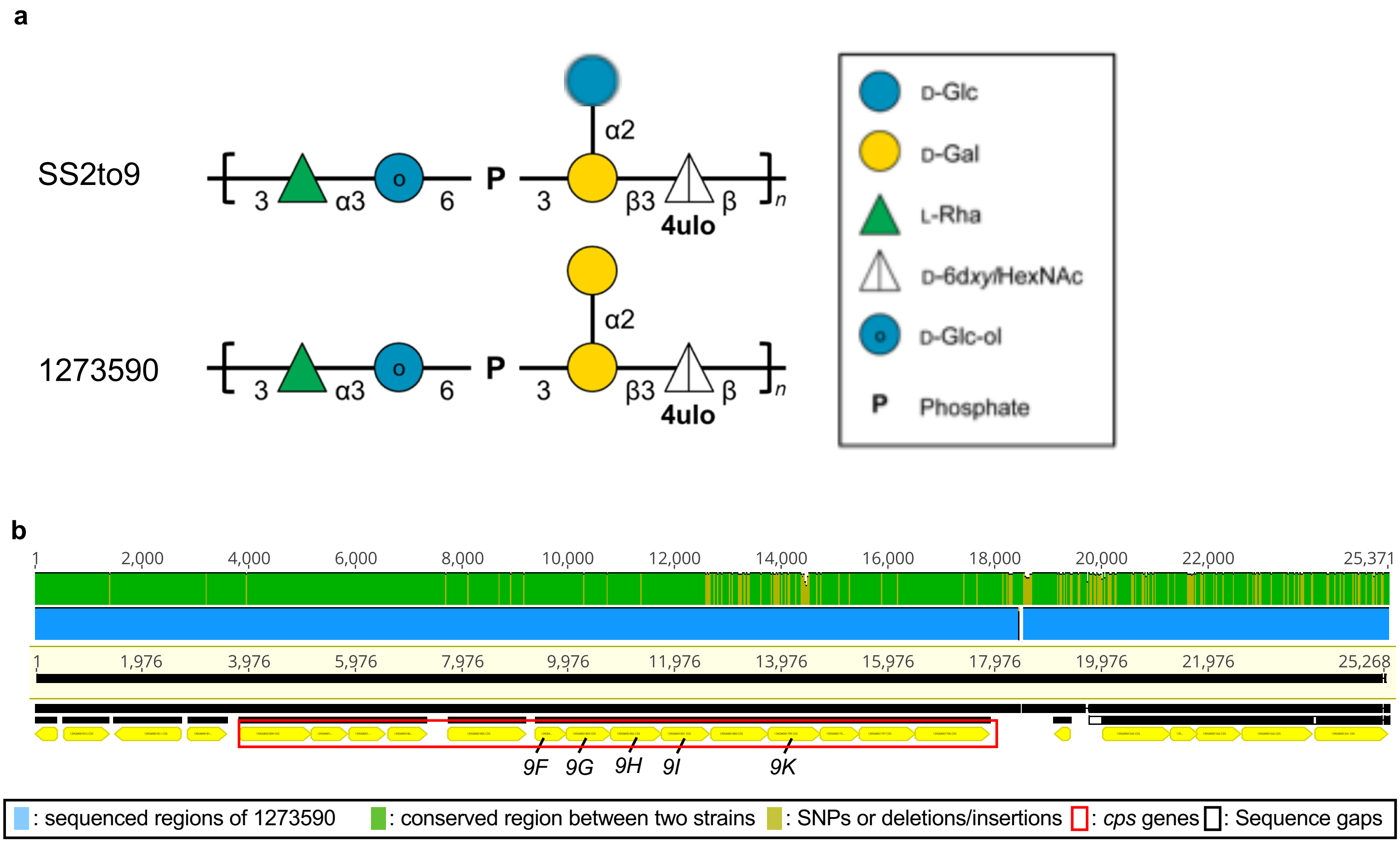

Fig S6. Difference in CPS between SS2to9 and strain 1273590. (**a**) Composition and structure of CPSs. (**b**) Nucleotide sequence alignment between the *cps* loci. Each schematic representation shows the analysis data using Geneious Prime. Below the bottom panel are displayed the descriptions for each color of the different drawings. Nucleotide sequence of *cps* locus of 1273590 was extracted from its draft genome sequence (Accession no. SRS1751390). Glycosyl transferase genes, *cps9F*, *cps9G*, *cps9H*, *cps9I*, and *cps9K* were appended.

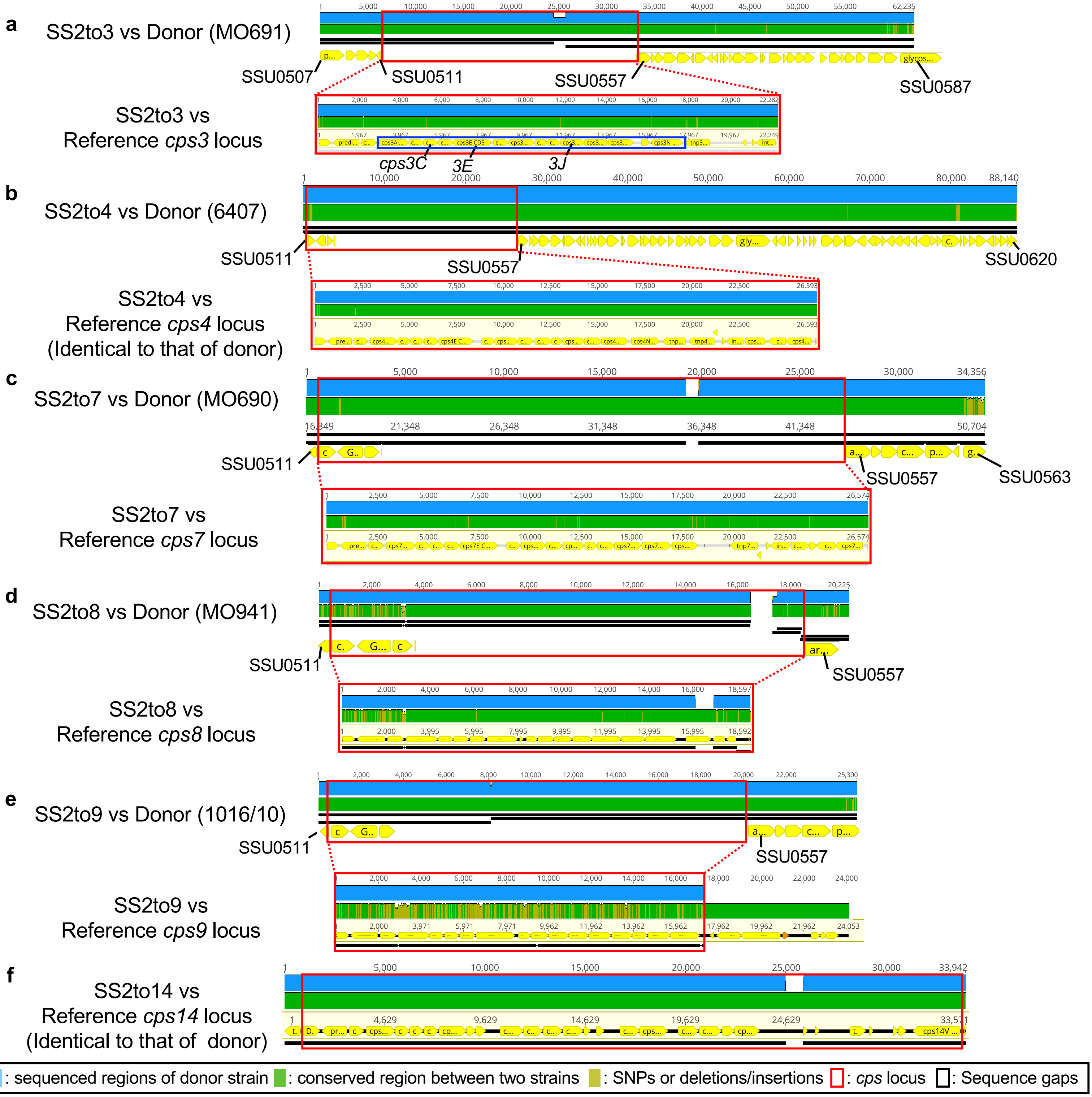

Fig S7. Replacement between *cps* loci. (**a**) SS2to3, (**b**) SS2to4, (**c**) SS2to7, (**d**) SS2to8, (**e**) SS2to9, and (**f**) SS2to14. Each schematic representation shows the analysis data using Geneious Prime on the sequence alignment between the *cps* loci and their flanking regions of the serotype-switched mutants and donor strains (upper part) and between the *cps* loci of the serotype-switched mutants and reference serotype strains (lower part). Below the bottom panel are displayed the descriptions for each color of the different drawings. .

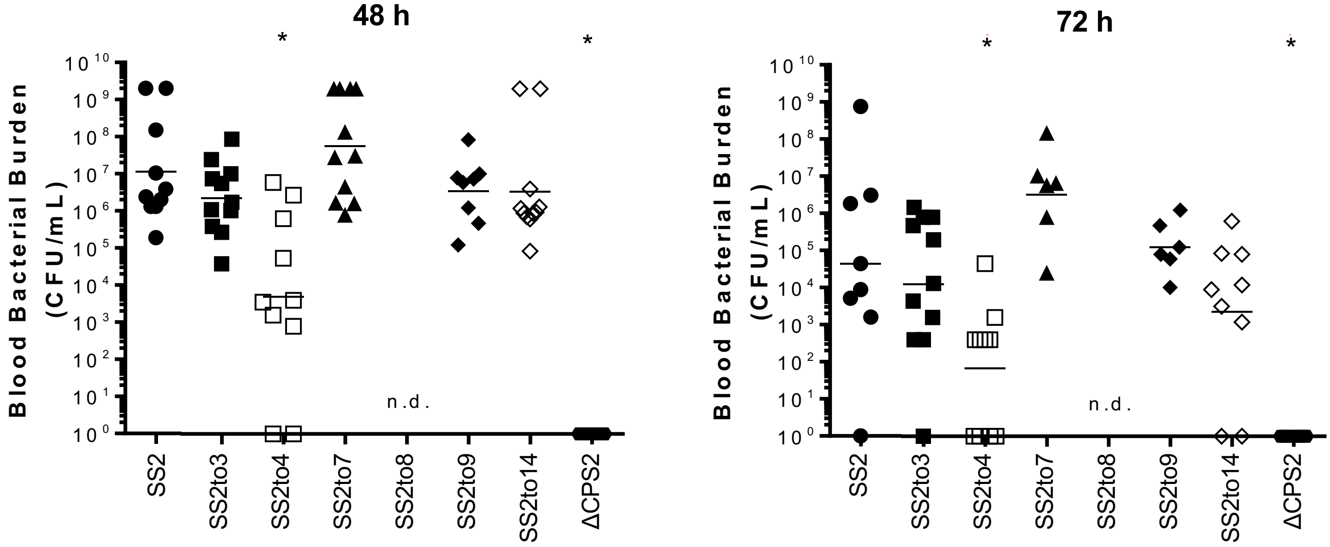

Fig S8. Blood bacterial burdens at 48 h and 72 h post-infection of mice inoculated with the different *S. suis* strains and mutants. Data represent the geometric mean (n = 10-12). A blood bacterial burden of 2 × 10^9 CFU/mL, corresponding to average burden upon euthanasia, was attributed to euthanized mice. n.d. denotes not determined. An asterisk denotes a significant difference with SS2 by Mann-Whitney rank sum test ($p < 0.05$).
