# Supplementary material for: Capsular polysaccharide switching in Streptococcus suis modulates host cell interactions and virulence
Source: Sci Rep. 2021 Mar 22;11:6513. doi: 10.1038/s41598-021-85882-3 (PMC7985379; doi:10.1038/s41598-021-85882-3)
Supplement: Supplementary file 2 — Supplementary Information 1. [file 41598_2021_85882_MOESM2_ESM.docx]

**Supplementary Methods**

**Capsular polysaccharide switching in *Streptococcus* *suis* modulates host cell interactions and virulence**

Masatoshi Okura^1#*^, Jean-Philippe Auger^2#^, Tomoyuki Shibahara^3,4#^, Guillaume Goyette-Desjardins^2^, Marie-Rose Van Calsteren^5^, Fumito Maruyama^6,7^, Mikihiko Kawai^8^, Makoto Osaki^1^, Mariela Segura^2*^, Marcelo Gottschalk^2*^, Daisuke Takamatsu^1,9^

^1^Division of Bacterial and Parasitic Diseases, National Institute of Animal Health, National Agriculture and Food Research Organization, Tsukuba, Ibaraki, Japan

^2^Faculty of Veterinary Medicine, University of Montreal, Saint-Hyacinthe, Quebec, Canada

^3^Division of Pathology and Pathophysiology, National Institute of Animal Health, National Agriculture and Food Research Organization, Tsukuba, Ibaraki, Japan

^4^Department of Veterinary Science, Graduate School of Life and Environmental Sciences, Osaka Prefecture University, Izumisano, Osaka, Japan

^5^Saint-Hyacinthe Research and Development Centre, Agriculture and Agri-Food Canada, Saint-Hyacinthe, Quebec, Canada

^6^Microbial Genomics and Ecology, Office of Industry-Academia-Government and Community Collaboration, Hiroshima University, Hiroshima, Japan

^7^Scientific and Technological Bioresource Nucleus, Universidad de La Frontera, Temuco, Chile

^8^Graduate School of Human and Environmental Studies, Kyoto University, Kyoto, Japan

^9^The United Graduate School of Veterinary Sciences, Gifu University, Gifu, Gifu, Japan

^#^These authors contributed equally to this work

*****Co-corresponding authors: E-mail: mokura@affrc.go.jp (M. Okura); marcelo.gottshcalk@umontreal.ca (M. Gottschalk); mariela.segura@umontreal.ca (M. Segura)

**Construction of the markerless non-encapsulated mutant**

Blue-white screening method using 5-bromo-4-chloro-3-indoxyl-α-L-fucopyranoside (X-α-L-fucopyranoside) was developed for construction of the markerless non-encapsulated mutant (**Fig. S3**). X-α-L-fucopyranoside is a chromogenic substrate for α-L-fucosidase, producing a blue precipitate; therefore, strains producing this enzyme form blue colonies on agar media supplemented with X-α-L-fucopyranoside. Our previous genome analysis data of 57 *S. suis* strains showed that 19 strains including MNCM50 had α-L-fucosidase gene (*afuC*), but that the other 38 strains including P1/7 did not [M1]. This indicates that X-α-L-fucopyranoside and the *afuC* gene can be used for screening of mutants in *S. suis*. Indeed, P1/7, which forms white colonies on TH agar supplemented with X-α-L-fucopyranoside (GoldBio, Olivette, MO, USA), formed blue colonies on the agar when the entire *afuC* gene of MNCM50 was introduced by pMX1-based expression vector (pAfuC) [M2]. For constructing the markerless non-encapsulated mutant from P1/7 using this tool, the DNA fragment composed of the *afuC*-*cat* cassette flanked by approximately 1 kbp of the upstream and downstream regions of the *cps2* gene cluster of P1/7 was then generated by overlap-extension PCR. The plasmids pAfuC and pSET1 were used as templates for amplification of the *afuC* and *cat*, respectively. The DNA fragment (at a final concentration of 5 ng/µL) was introduced into *S. suis* strain P1/7 with XIP as described above. The transformants (ΔCPS2toafuCcat), in which the *cps* gene cluster was replaced with the *afuC*-*cat* cassette, were selected on TH plates with CP. A DNA fragment only composed of about 1 kbp of the upstream and downstream regions of the *cps2* gene cluster of P1/7 without the cluster was transformed into ΔCPS2toafuCcat with XIP. The transformed culture was diluted 10^3^, 10^4^, and 10^5^ times, and the dilutions were spread on 10 TH agar plates containing 100 µg/mL of X-α-L-fucopyranoside. After incubation for 1-2 days at 37°C in a 5% CO_2_-enriched atmosphere, white colonies, from which the *afuC*-*cat* cassette was considered to be lost, were selected and subcultured on TH agar plate (frequency of transformants was approximately 10^-4^~10^-5^ when flanking regions of 1kb is used). Deletion of the *afuC*-*cat* cassette in the selected transformants was confirmed by CP susceptibility and PCR, and one of the markerless non-encapsulated mutants designated as ΔCPS2 was used in this study.

**Detail method on confirmation of serotype switching**

1. **Serotyping**

Anti-serotype 3, 4, 7, 8, 9, and 14 sera were purchased from Statens Serum Institut (Copenhagen, Denmark), whereas anti-serotype 2 serum used in this study was prepared previously [M3] by immunizing rabbits with formalin-killed *S. suis* strain NCTC 10234^T^ according to the previously described procedure [M4]. Depending on the sensibility of the specific CPS to hydrolysis, two methods of extraction of the capsular antigens were used. The capsular antigens of tested strains/mutants were extracted by autoclaving in Dulbecco’s Phosphate-Buffered Saline [(D-PBS) FUJIFILM-Wako Pure Chemical Corp., Tokyo, Japan] for serotypes 2, 3, 4, and 14 or by heating in deionized water (ddH_2_O) at 60°C for 45 min for serotypes 7, 8, and 9. The autoclaved and heat-treated bacterial suspensions were allowed to cool, and the supernatants collected by centrifugation were used as antigens. The co-agglutination technique was applied as previously described [M3]. For further confirmation for serotype 4, the Ouchterlony double immunodiffusion assay was carried out. Four microliters of each antigen were added to wells cut equidistant from each other in 1% agarose (Difco™ Noble Agar; Becton Dickinson) in D-PBS solution containing 0.1% sodium azide. These antigens were allowed to diffuse against 15 µL of the tested antiserum in the central well for 24 h at 4°C.

1. **Hydrophobicity assay**

Surface hydrophobicity of *S. suis* non-encapsulated strains has been demonstrated to be significantly higher than that of encapsulated strains [M5]. To verify CPS expression, cell surface hydrophobicity tests were carried out by measuring their absorption to *n*-hexadecane as previously described [M5]. The serotype 2 P1/7 strain and non-encapsulated mutant ΔCPS2 were used as negative and positive controls, respectively.

1. **Transmission electron microscopy**

TEM was carried out to confirm CPS expression at the bacterial surface of serotype-switched mutant strains as previously described [M3], with some modifications. Briefly, bacteria were grown to mid-logarithmic phase in TH broth, washed with D-PBS, and incubated with anti-serum specific for each of the serotypes at 4°C for 1 h. The cells were then washed with 0.1 M cacodylate buffer (pH 7.3) and fixed with 0.1 M cacodylate buffer (pH 7.3) containing 5% (v/v) glutaraldehyde and 0.15% (w/v) ruthenium red for 2 h at room temperature. The cells were immobilized using iPGell (GenoStaff, Tokyo, Japan) and post-fixed with 1% (v/v) osmium tetroxide in water for 2 h at room temperature. Samples were washed thrice with 70% ethanol for 15 min to remove osmium tetroxide and dehydrated in an increasing graded series of ethanol. Specimens were then washed twice in methylglycidyl ether (QY-2; Nisshin EM, Tokyo, Japan) and embedded in low-viscosity resin (Quetol 651 mixture; Nisshin EM). Ultrathin sections were post-stained with uranyl acetate and lead citrate prior to examination with a transmission electron microscope at 80 kV (model H-7500; Hitachi, Tokyo, Japan).

1. ***S. suis* CPS purification**

The CPS from the different serotype-switched mutants were purified as previously described [M6, M7]. Briefly, 6 L of fresh TH broth were inoculated with the appropriate strain and incubated overnight. Depending on the sensibility of the specific CPS to hydrolysis, two methods of CPS extraction were used. For the SS2to3 and SS2to14 mutants, the autoclave method was used [M6, M8]. Briefly, bacterial cells from the 6 L cultures were pelleted by centrifugation at 10,000 × *g* for 40 min, resuspended by repeated pipetting in a buffer containing 33 mM phosphate and 145 mM NaCl, pH 8.0, and chilled. The bacterial suspension was autoclaved at 121°C for 15 min. The supernatant containing the crude CPS was recovered by centrifugation at 9,000 × *g* for 50 min. For the SS2to7, SS2to8, and SS2to9 mutants, the water method was used [M7, M9]. Briefly, the bacterial pellet from the 6 L was resuspended in ddH_2_O. Bacteria were killed by heating at 60°C for 45 min, which was confirmed by the absence of growth on blood agar plates. Finally, the content of the tubes was lyophilized for 72 h. Bacteria were then resuspended with ddH_2_O (3 g of dry cells in 100 mL of ddH_2_O) overnight at room temperature. The supernatant containing the crude CPS was recovered by centrifugation at 9,000 × *g* for 50 min. The crude CPSs were further purified from the extracted material by solvent extraction, precipitations, and gel filtration chromatography as previously described [M9]. For gel filtration chromatography, a XK-26/100 column packed with Sephacryl S-400 HR (GE Healthcare Life Sciences, Uppsala, Sweden) was used and eluted with 50 mM NH_4_HCO_3_ (for the SS2to3 and SS2to14 mutants) or with 50 mM NaCl (for the SS2to7, SS2to8, and SS2to9 mutants) at a flow rate of 1.3 mL/min, using an ÄKTA Purifier 10 system (GE Healthcare Life Sciences), including a UV-900 Monitor, and equipped with a Knauer Smartline 2300 RI Detector (Knauer, Berlin, Germany) connected to the system via an AD-900 Analog/Digital Converter (GE Healthcare Life Sciences). Fractions giving a positive RI signal but no absorption at 280 nm and 254 nm were pooled and lyophilized. The purified material was dissolved in water, dialyzed against ddH_2_O for 24 h at 4°C, and finally lyophilized.

1. **Nuclear magnetic resonance (NMR) spectroscopy**

Purified CPSs from SS2to3 and SS2to14 were exchanged in phosphate buffer pH 8.0 in D_2_O (99.9 atom% D), lyophilized, and dissolved in D_2_O (99.96 atom% D) to a final concentration of 33 mM, whereas CPSs from SS2to7, SS2to8, and SS2to9 were exchanged in D_2_O (99.9 atom% D), lyophilized, and dissolved in D_2_O (99.96 atom% D). NMR spectra were acquired on CPS samples at concentrations of 0.6–1.0% at 11.75 T using a Bruker Avance 500 spectrometer equipped with a 5 mm triple resonance TBI probe with ^1^H, ^13^C, and ^109^Ag–^31^P channels at 25–85°C using standard Bruker pulse sequences at the Centre régional de résonance magnétique nucléaire (Department of Chemistry, University of Montreal). For conventional ^1^H spectra, 32 K complex data points were acquired after a 30° pulse with a digital resolution of 0.18 Hz/point and processed off-line using SpinWorks version 4.2.8.0 (Copyright © Kirk Marat, home.cc.umanitoba.ca/~wolowiec/spinworks/) by exponential multiplication with a 0.2 Hz line broadening factor, zero filling, complex Fourier transform, phase correction, and fifth-order polynomial baseline correction. ^1^H chemical shifts δ in ppm were referenced to internal deuterated 2,2-dimethyl-2-silapentane-5-sulfonate (DSS-*d*_6_) at δ 0 as recommended by Wishart et al. [M10].

**Whole genome sequencing and sequence comparison**

Whole genome draft sequences of strain P1/7, ΔCPS2tocat, three strains used as DNA donors (MO690, MO691, and MO941), and six serotype-switched mutants (SS2to3, SS2to4, SS2to7, SS2to8, SS2to9, and SS2to14) were determined. Genomic DNA was extracted using the DNeasy Blood and Tissue Kit (QIAGEN) and paired-end libraries with an average insert size of 350 bp were prepared, followed by 2 × 150 bp sequencing on Illumina HiSeq X ten sequencing platform at the Beijing Genomics Institute (Shenzhen, China) or Illumina NovaSeq platform at Novogene Corporation (San Diego, CA, USA) (only for SS2to9). Except for SS2to9, quality trimming and adapter trimming were conducted using Cutadapt (https://github.com/marcelm/cutadapt/) via TrimGalore (https://github.com/FelixKrueger/TrimGalore). Mismatch correction of reads and assembly were carried out using SPAdes [M11], and the assembly was polished using Pilon [M12], with the aid of Unicycler [M13]. The completeness and contamination of the final draft genome sequence were assessed using CheckM [M14]. CheckM reported 100% completeness with 0% potential contamination for the final draft genome sequence. Regarding SS2to9, quality control of paired-end reads was performed using an in-house program and the reads were assembled using SOAP de novo software at Novogene Corporation. Calculations of ANI and a fraction shared between genome pairs were conducted using FastANI [M15].

**Detail methods on *in vitro* assays for evaluation of impacts on serotype switching**

**NPTr epithelial cell culture and adhesion and invasion assays**

The porcine tracheal epithelial NPTr cell line was used and cultured until confluence as previously described [M16]. Cells were infected with 1 × 10^6^ CFU/well [multiplicity of infection (MOI) = 10] of the different *S. suis* strains and incubated for 2 h at 37°C in 5% CO_2_. The adhesion assay, which quantifies total cell-associated bacteria (surface-adherent and intracellular bacteria), and invasion assay (using the antibiotic protection assay) were performed as previously described [M16].

**J774A.1 macrophage culture and phagocytosis assays**

J774A.1 murine macrophages (ATCC TIB-67; Rockville, MD, USA) were maintained in Dulbecco’s Modified Eagle’s Medium (Gibco, Burlington, ON, Canada) supplemented with 10% fetal bovine serum (Gibco) and grown at 37 °C with 5% CO_2_. Confluent cell cultures were scraped, seeded at 1 × 10^5^ cells/mL, and incubated for 3 h at 37 °C with 5% CO_2_ to allow cell adhesion. Cells were infected by adding 1 × 10^7^ CFU/mL of bacterial suspension in complete culture medium (MOI = 100), incubated for 1 h or 2 h at 37 °C with 5% CO_2_, and phagocytosis assays were performed as previously described using the antibiotic protection assay [M17].

**Murine whole blood bactericidal assay**

Blood was collected from 6- to 10-week-old C57BL/6J mice (Jackson Research Laboratories, Bar Harbor, ME, USA) and mixed with sodium heparin (Sigma-Aldrich, Oakville, ON, Canada). Blood (containing approximately 9 × 10^6^ leukocytes/mL) was transferred to a microtube containing 9 × 10^6^ CFU/mL of the *S. suis* strains (MOI = 1) and incubated for 4 h, mixing every 20 min. Assay conditions were chosen based on the kinetics of *S. suis* killing by murine blood [M17]. After incubation, cells were lysed by vortexing, and appropriate dilutions were plated on TH agar to determine viable bacterial counts. Resistance to bacterial killing by blood leukocytes was compared to incubation of the different strains in plasma only (obtained by centrifuging whole blood at 1,800 × *g* for 10 min at 4°C). Percentage of bacterial survival was calculated using the following formula: (bacteria in blood / bacteria in plasma) × 100%.

**Porcine whole blood bactericidal assay**

Porcine blood killing assays were performed as previously described with some modifications [M18]. Briefly, blood was collected from a 6-week-old piglet and mixed with sodium heparin (Sigma-Aldrich). The animal did not have any episode of acute disease related to *S. suis*. Eighty microliters of each of the strains, grown at logarithmic phase in TH broth (2 × 10^6^ CFU/mL) was mixed with 160 µL of D-PBS or heparinized blood (containing approximately 1 × 10^8^ leukocytes/mL; MOI = 0.01) and incubated at 37°C for 4 h with gentle rotation. Bacterial capacity to grow in whole blood for 4 h incubation was compared to incubation at 0 h in D-PBS. The obtained CFUs were determined by plating serial dilutions on TH agar. Fold changes in viable bacteria in blood were defined by dividing the CFUs in the sample with blood by those with D-PBS.

**Generation of bone marrow-derived dendritic cells (DCs) and pro-inflammatory mediator production measurement**

Generation of bone marrow-derived DCs were performed as previously described [M19] using the femur and tibia of C57BL/6J mice (Jackson Research Laboratories). Briefly, hematopoietic bone marrow stem cells were cultured in Roswell Park Memorial Institute-1640 medium (Gibco) supplemented with 5% heat-inactivated fetal bovine serum, 10 mM 4-(2-hydroxyethyl)-1-piperazineethanesulfonic acid (Gibco), 2 mM l-glutamine (Gibco), and 50 μM 2-mercaptoethanol (Gibco). Complete medium was complemented with 20% granulocyte-macrophage colony-stimulating factor from mouse-transfected Ag8.653 cells [M20]. Prior to infection, cells were plated at 1 × 10^6^ cells/mL. Cell purity was determined to be at least 90% CD11c^+^ by flow cytometry. Cells were stimulated with the different *S. suis* strains in culture medium (1 × 10^6^ CFU/mL; initial MOI = 1) for 16 h, and supernatants were collected for the measurement of secreted levels of TNF, IL-6, IL-12p70, CCL2, CCL5, CXCL1, and CXCL9. Non-infected cells served as negative controls. Secreted levels of these mediators were quantified by sandwich ELISA using pair-matched antibodies from R&D Systems (Minneapolis, MN, USA) according to the manufacturer's recommendations.

**Detail methods on *in vivo* assays for evaluation of impacts on serotype switching**

***S. suis* mouse infection model**

Six-week-old male and female C57BL/6J mice (Jackson Research Laboratories) were used. Mice were acclimatized to standard laboratory conditions with unlimited access to water and rodent chow [M17]. *S. suis* strains were intraperitoneally inoculated at a dose of 1 × 10^7^ CFU to groups of 10-12 mice for survival and blood bacterial burden evaluation. Mice were monitored at least three times daily until 72 h post-infection and twice thereafter until 14 days post-infection. Blood bacterial burden of surviving mice was assessed 24 h, 48 h, and 72 h post-infection by collecting 5 µL of blood from the caudal tail vein, appropriately diluting and plating on TH agar.

**Measurement of plasma (systemic) pro-inflammatory mediators**

For measurement of plasma (systemic) pro-inflammatory mediators, eight mice were intraperitoneally infected with 1 × 10^7^ CFU of the different *S. suis* strains. Blood was collected 12 h post-infection by intracardiac puncture and anti-coagulated with EDTA (Sigma-Aldrich) as previously described [M17]. Plasma supernatants were collected following centrifugation at 10,000 × *g* for 10 min at 4°C and stored at −80°C. This time point was selected to obtain maximal pro-inflammatory mediator production in the absence of significant mouse mortality. Plasmatic concentrations of IL-6, IL-12p70, IFN-γ, CCL2, CCL3, CCL4, CCL5, and CXCL2 were measured using a custom-made cytokine Bio-Plex Pro™ assay (Bio-Rad) according to the manufacturer's instructions. These mediators were selected based on serotype 2 studies and represent the most important pro-inflammatory cytokines and chemokines secreted [M17]. The data were acquired from the MAGPIX platform (Luminex®) and analyzed using the Bio-Plex Manager 6.1 software (Bio-Rad).

***S. suis* porcine infection model**

Crossbred male and female piglets aged of 5 weeks, age at which the piglets are most susceptible to *S. suis* infection in the field [M21], were purchased from Shokukanken Inc. (Gunma, Japan) and CIMCO Co. Ltd. (Tokyo, Japan). These pigs were negative for pathogens responsible for porcine reproductive and respiratory syndrome, pseudorabies, porcine epidemic diarrhea, transmissible gastroenteritis, atrophic rhinitis, porcine enzootic pneumonia, swine dysentery, salmonellosis, toxoplasmosis, and actinobacillosis. Pigs were housed in a closed animal facility and received antibiotic-free diets (FEED ONE, Yokohama, Japan). Before infection, pigs were confirmed to be negative for *S. suis* serotypes 2, 3, 4, 7, 8, and 14 by *cps* type specific PCR [M22] with DNA extracted from the culture of nasal swabs in TH broth overnight at 37°C. Infections were carried out as previously described [M23] and divided into two experiments: in the first experiment, 16 pigs (Shokukanken Inc.) were allocated into four groups and infected with SS2 (Group I-1; n = 4), ΔCPS2 (Group I-2; n = 4), SS2to4 (Group I-3; n = 4) or SS2to7 (Group I-4; n = 4); in the second experiment, 17 pigs (CIMCO Co. Ltd.) were divided into four groups and infected with SS2 (Group II-1; n = 4), SS2to3 (Group II-2; n = 4), SS2to8 (Group II-3; n = 4), or SS2to14 (Group II-4; n = 5). Five milliliters of 1% acetic acid (pH 3.0, FUJIFILM-Wako Pure Chemical Corp.) was intranasally administered to each pig, an irritant of the upper respiratory tract used for *S. suis* experimental infections [M23, M24], using a disposable nasal spray device (TOP Corp., Tokyo, Japan). One hour later, pigs were inoculated with 1 mL of nasal spray containing approximately 2 × 10^9^ CFU/mL of the different *S. suis* strains. Pigs were monitored for clinical signs of nervous, musculoskeletal, or respiratory disease, and scored based on body temperature (anal temperature), attitude, and locomotion for 7 days post-inoculation (dpi) [M25] (**Table SM1 below**). Pigs having a clinical score >2 on attitude or locomotion were euthanized by lethal injection for ethical reasons. All surviving pigs were sacrificed at 7 dpi. During necropsy, brain, lung, spleen, liver, heart, joint, tonsil, and blood samples were collected and cultured for bacterial recovery. Isolation of the infected strains was confirmed by PCR assay for detection of *cps* genes specific for each serotype (for SS2 and serotype-switched mutants) and deletion in *cps* gene cluster (for ΔCPS2).

**Table SM1. Score values for body temperature, attitude and locomotion.**

| **Score values** | **Body temperature** | **Attitude** | **Locomotion** |
| --- | --- | --- | --- |
| 0 | <40.4°C | normal attitude and response to stimuli | normal gait and posture |
| 1 | 40.5-40.9°C | inactive and slow to respond with oculonasal secretions | slight incoordination, lameness, and/or joint swelling but rises without assistance |
| 2 | 41-41.4°C | only responsive to repeated stimuli | clearly uncoordinated or lame but stands without assistance |
| 3 | 41.5-41.9°C | recumbent, nonresponsive, and unaware of surroundings | severe lameness and/or severe ataxia |
| 4 | >42°C | dead | dead |

**Reference**

M1. Okura M, Nozawa T, Watanabe T, Murase K, Nakagawa I, Takamatsu D, Osaki M, Sekizaki T, Gottschalk M, Hamada S, Maruyama F. 2017. A locus encoding variable defense systems against invading DNA identified in *Streptococcus suis*. Genome Biol Evol. 9: 1000–1012. doi: 10.1093/gbe/evx062.

M2. Okura M, Osaki M, Fittipaldi N, Gottschalk M, Sekizaki T, Takamatsu D. 2011. The minor pilin subunit Sgp2 is necessary for assembly of the pilus encoded by the *srtG* cluster of *Streptococcus suis*. J Bacteriol. 193:822–831. doi: 10.1128/JB.01555-09.

M3. Lakkitjaroen N, Takamatsu D, Okura M, Sato M, Osaki M, Sekizaki T. 2011. Loss of capsule among *Streptococcus suis* isolates from porcine endocarditis and its biological significance. J Med Microbiol. 60: 1669–1676. doi: 10.1099/jmm.0.034686-0.

M4. Higgins R, Gottschalk M. 1990. An update on *Streptococcus suis* identification. J Vet Diagn Invest. 2:249–252. doi: 10.1177/104063879000200324.

M5. Bonifait L, Gottschalk M, Grenier D. 2010. Cell surface characteristics of nontypeable isolates of *Streptococcus suis*. FEMS Microbiol Lett. 311:160–166. doi: 10.1111/j.1574-6968.2010.02086.x.

M6. Goyette-Desjardins G, Vinogradov E, Okura M, Takamatsu D, Gottschalk M, Segura M. 2018. *Streptococcus suis* serotype 3 and serotype 18 capsular polysaccharides contain di-*N*-acetyl-bacillosamine. Carbohydr Res 466: 18–29. doi: 10.1016/j.carres.2018.07.003.

M7. Goyette-Desjardins G, Vinogradov E, Okura M, Takamatsu D, Gottschalk M, Segura M. 2019. Structure determination of *Streptococcus suis* serotypes 7 and 8 capsular polysaccharides and assignment of functions of the *cps* locus genes involved in their biosynthesis. Carbohydr Res. 473:36–45. doi: 10.1016/j.carres.2018.12.009.

M8. Van Calsteren MR, Gagnon F, Calzas C, Goyette-Desjardins G, Okura M, Takamatsu D, Gottschalk M, Segura M. 2013. Structure determination of *Streptococcus suis* serotype 14 capsular polysaccharide. Biochem Cell Biol. 91:49–58. doi: 10.1139/bcb-2012-0036.

M9. Vinogradov E, Goyette-Desjardins G, Okura M, Takamatsu D, Gottschalk M, Segura M. 2016. Structure determination of *Streptococcus suis* serotype 9 capsular polysaccharide and assignment of functions of the *cps* locus genes involved in its biosynthesis. Carbohydr Res. 433:25–30. doi: 10.1016/j.carres.2016.07.005.

M10. Wishart DS, Bigam CG, Yao J, Abildgaard F, Dyson HJ, Oldfield E, Markley JL, Sykes BD. 1995. ^1^H, ^13^C and ^15^N chemical shifts referencing in biomolecular NMR. J Biomol NMR. 6: 135–140. doi: 10.1007/BF00211777.

M11. Nurk S, Bankevich A, Antipov D, Gurevich AA, Korobeynikov A, Lapidus A, Prjibelski AD, Pyshkin A, Sirotkin A, Sirotkin Y, Stepanauskas R, Clingenpeel SR, Woyke T, McLean JS, Lasken R, Tesler G, Alekseyev MA, Pevzner PA. 2013. Assembling single-cell genomes and mini-metagenomes from chimeric MDA products. J Comput Biol. 20:714–737. doi: 10.1089/cmb.2013.0084.

M12. Walker BJ, Abeel T, Shea T, Priest M, Abouelliel A, Sakthikumar S, Cuomo CA, Zeng Q, Wortman J, Young SK, Earl AM. 2014. Pilon: an integrated tool for comprehensive microbial variant detection and genome assembly improvement. PLoS One. 9:e112963. doi: 10.1371/journal.pone.0112963.

M13. Wick RR, Judd LM, Gorrie CL, Holt KE. 2017. Unicycler: Resolving bacterial genome assemblies from short and long sequencing reads. PLoS Comput Biol. 13:e1005595. doi: 10.1371/journal.pcbi.1005595.

M14. Parks DH, Imelfort M, Skennerton CT, Hugenholtz P, Tyson GW. 2015. CheckM: assessing the quality of microbial genomes recovered from isolates, single cells, and metagenomes. Genome Res. 25:1043–1055. doi: 10.1101/gr.186072.114.

M15. Jain C, Rodriguez RL, Phillippy AM, Konstantinidis KT, Aluru S. 2018. High throughput ANI analysis of 90K prokaryotic genomes reveals clear species boundaries. Nat Commun. 9:5114. doi: 10.1038/s41467-018-07641-9.

M16. Wang Y, Gagnon CA, Savard C, Music N, Srednik M, Segura M, Lachance C, Bellehumeur C, Gottschalk M. 2013. Capsular sialic acid of *Streptococcus suis* serotype 2 binds to swine influenza virus and enhances bacterial interactions with virus-infected tracheal epithelial cells. Infect Immun. 81: 4498–4508. doi: 10.1128/IAI.00818-13.

M17. Auger JP, Fittipaldi N, Benoit-Biancamano MO, Segura M, Gottschalk M. 2016. Virulence studies of different sequence types and geographical origins of *Streptococcus suis* serotype 2 in a mouse model of infection. Pathogens. 5:E48. doi: 10.3390/pathogens5030048.

M18. Auger JP, Payen S, Roy D, Dumesnil A, Segura M, Gottschalk M 2019. Interactions of *Streptococcus suis* serotype 9 with host cells and role of the capsular polysaccharide: Comparison with serotypes 2 and 14. PLoS One. 14:e0223864. doi: 10.1371/journal.pone.0223864.

M19. Lecours MP, Gottschalk M, Houde M, Lemire P, Fittipaldi N, Segura M. 2011. Critical role for *Streptococcus suis* cell wall modifications and suilysin in resistance to complement-dependent killing by dendritic cells. J Infect Dis. 204: 919–929. doi: 10.1093/infdis/jir415.

M20. Segura M, Su Z, Piccirillo C, Stevenson MM. 2007. Impairment of dendritic cell function by excretory-secretory products: a potential mechanism for nematode-induced immunosuppression. Eur J Immunol. 37:1887–1904. doi: 10.1002/eji.200636553.

M21. Gottschalk M. 2012. Streptococcosis. p 841–855. *In* Zimmerman JJ, Karriker LA, Ramirez A, Schwartz KJ, Stevenson GW (ed), Diseases of Swine, 10th ed, Wiley-Blackwell, Ames, IA.

M22. Okura M, Lachance C, Osaki M, Sekizaki T, Maruyama F, Nozawa T, Nakagawa I, Hamada S, Rossignol C, Gottschalk M, Takamatsu D. 2014. Development of a two-step multiplex PCR assay for typing of capsular polysaccharide synthesis gene clusters of *Streptococcus suis*. J Clin Microbiol. 52:1714–1719. doi: 10.1128/JCM.03411-13.

M23. Pallarés FJ, Halbur PG, Schmitt CS, Roth JA, Opriessnig T, Thomas PJ, Kinyon JM, Murphy D, Frank DE, Hoffman LJ. 2003. Comparison of experimental models for *Streptococcus suis* infection of conventional pigs. Can J Vet Res. 67:225–228.

M24. Baums CG, Kaim U, Fulde M, Ramachandran G, Goethe R, Valentin-Weigand P. 2006. Identification of a novel virulence determinant with serum opacification activity in *Streptococcus suis*. Infect Immun. 74: 6154–6162. doi: 10.1128/IAI.00359-06.

M25. Li Y, Gottschalk M, Esgleas M, Lacouture S, Dubreuil JD, Willson P, Harel J. 2007. Immunization with recombinant Sao protein confers protection against *Streptococcus suis* infection. Clin Vaccine Immunol. 14:937–943. doi: 10.1128/CVI.00046-07.
