# Supplementary material for: Capsular polysaccharide switching in Streptococcus suis modulates host cell interactions and virulence
Source: Sci Rep. 2021 Mar 22;11:6513. doi: 10.1038/s41598-021-85882-3 (PMC7985379; doi:10.1038/s41598-021-85882-3)
Supplement: Supplementary file 3 — Supplementary Information 2. [file 41598_2021_85882_MOESM3_ESM.docx]

**Supplementary Notes**

**Capsular polysaccharide switching in *Streptococcus* *suis* modulates host cell interactions and virulence**

Masatoshi Okura^1#*^, Jean-Philippe Auger^2#^, Tomoyuki Shibahara^3,4#^, Guillaume Goyette-Desjardins^2^, Marie-Rose Van Calsteren^5^, Fumito Maruyama^6,7^, Mikihiko Kawai^8^, Makoto Osaki^1^, Mariela Segura^2*^, Marcelo Gottschalk^2*^, Daisuke Takamatsu^1,9^

^1^Division of Bacterial and Parasitic Diseases, National Institute of Animal Health, National Agriculture and Food Research Organization, Tsukuba, Ibaraki, Japan

^2^Faculty of Veterinary Medicine, University of Montreal, Saint-Hyacinthe, Quebec, Canada

^3^Division of Pathology and Pathophysiology, National Institute of Animal Health, National Agriculture and Food Research Organization, Tsukuba, Ibaraki, Japan

^4^Department of Veterinary Science, Graduate School of Life and Environmental Sciences, Osaka Prefecture University, Izumisano, Osaka, Japan

^5^Saint-Hyacinthe Research and Development Centre, Agriculture and Agri-Food Canada, Saint-Hyacinthe, Quebec, Canada

^6^Microbial Genomics and Ecology, Office of Industry-Academia-Government and Community Collaboration, Hiroshima University, Hiroshima, Japan

^7^Scientific and Technological Bioresource Nucleus, Universidad de La Frontera, Temuco, Chile

^8^Graduate School of Human and Environmental Studies, Kyoto University, Kyoto, Japan

^9^The United Graduate School of Veterinary Sciences, Gifu University, Gifu, Gifu, Japan

^#^These authors contributed equally to this work

*****Co-corresponding authors: E-mail: mokura@affrc.go.jp (M. Okura); marcelo.gottshcalk@umontreal.ca (M. Gottschalk); mariela.segura@umontreal.ca (M. Segura)

**Difference in the side chain of the CPS repeating unit between the serotype 9 and serotype 9 variant strains**

The nucleotide sequences of all *cps* genes were identical between SS2to9 and the donor strain 1016/10 (**Fig. S8E**), whereas disagreement of nucleotide sequence was found in five *cps* genes between the two strains and strain 1273590 (**Fig. S6B and Table SN-1 below**) Disagreement of nucleotide sequence was found in five glycosyl transferase genes (*cps9F*, *cps9G*, *cps9H*, *cps9I*, and *cps9K*) and a repeating unit polymerase gene (*cps9J*) between them and strain 1273590. A glycosyl transferase gene *cps9H* was predicted to be associated with transfer of the galactose to form this side chain [N1] based on similarity with *wcxS* of *Streptococcus pneumoniae* type 45, a gene later reassigned to the transfer of a rhamnose (l-Rha*p*) donor forming an α-(1→3) linkage in types 45 and 16A [N2]; however, only one synonymous single nucleotide polymorphism was found in *cps9H* between the two *S. suis* strains. Among the glycosyl transferase genes in the *cps9* locus, nonsynonymous single nucleotide polymorphisms were found only in *cps9I* and *cps9K*. These nucleotide sequence differences in the *cps9I* and *cps9K* caused 2 and 20 amino acid substitutions, respectively. These results suggest that, similarly to the cases of serotypes 1 and 14 and serotypes 2 and 1/2 [N3], transfer of side-chain galactose or glucose is probably dictated by differences in either *cps9I* or *cps9K.* It also remains unclear whether this difference in CPS affect the virulence of *S. suis*; the serotype 9 variant donor strain belongs to the same ST (ST16) as the serotype 9 strains frequently isolated in European countries [N4], and nucleotide sequence of *cps9* locus of SS2to9 is completely identical with that of a genome sequenced ST16 strain GD-0088 isolated from a diseased pig in the Netherlands (Accession no. LR738723), suggesting that the CPS structure of the ST16 strains is the same as that of mutant SS2to9.

**Table SN-1. Comparison of the sequences of *cps* genes between the mutant SS2to9 and serotype 9 strain 1273590**

|  | | | |  |  |
| --- | --- | --- | --- | --- | --- |
| **Locus tag** | **Product (putative)** | **Function/Pathway^a^** | **No. of disagreed nucleotides** | **Amino acids identity (%)** | |
| *cps9A* | Integral membrane regulatory protein Wzg | Regulation | 1 | 444/445 | 99.8 |
| *cps9B* | Chain length determinant protein/polysaccharide export protein Wzd | Regulation | 0 | 229/229 | 100.0 |
| *cps9C* | Tyrosine-protein kinase Wze | Regulation | 0 | 228/228 | 100.0 |
| *cps9D* | Protein-tyrosine phosphatase Wzh | Regulation | 0 | 243/243 | 100.0 |
| *cps9E* | Nucleoside-diphosphate sugar epimerase | 2-acetamido-2,6-dideoxy-D-*xylo*-hexose (D-6d-*xyl*HexNAc) synthesis? | 4 | 482/486 | 99.2 |
| *cps9F* | Initial sugar transferase | Transfer of D-6d-*xyl*HexNAc to a lipid carrier | 0 | 200/200 | 100.0 |
| *cps9G* | Glycosyltransferase | Transfer of galactose (Gal) to D-6d-*xyl*HexNAc? | 1 | 271/271 | 100.0 |
| *cps9H* | Glycosyltransferase | Transfer of Gal at the side chain to Gal? | 1 | 318/318 | 100.0 |
| *cps9I* | LicD-family protein | Transfer of glucitol (Glc-ol) phosphate to Gal? | 4 | 303/305 | 99.3 |
|  | *Thought to be a glycosyltransferase* |  |  |  |  |
| *cps9J* | Oligosaccharide repeat unit polymerase Wzy | Polymerase | 16 | 351/355 | 98.9 |
| *cps9K* | Capsular polysaccharide synthesis protein | Transfer of rhamnose to Glc-ol ? | 44 | 299/319 | 93.4 |
| *cps9L* | 2-C-methyl-D-erythritol 4-phosphate cytidylyltransferase | Glc-ol synthesis? | 2 | 237/239 | 99.2 |
| *cps9M* | NAD-dependent epimerase/dehydratase | Glc-ol synthesis? | 2 | 351/351 | 100.0 |
| *cps9N* | Flippase Wzx | Transport of repeat units | 2 | 463/463 | 100.0 |

a: Predicted in the previous study [N1]

**Comparison of the genomes between the serotype-switched mutants and the recipient serotype 2 strain**

As shown in Table S2, amino acid sequences of the encoded proteins of almost all the mutated genes showed more than 95% identity with those of the SS2 genome (**Table S2**). However, SS2to3 and its donor MO691 lost two genes related to the putative toxin-antitoxin system (SS2 corresponding locus tags SSU0571 and SSU0572) and possessed a putative membrane protein (SS2 corresponding locus tag SSU0568), the N-terminal of which was completely different from that of SS2 (**Table S2**). In the SS2to4 and SS2to9 genomes, amino acid sequences of six proteins (corresponding to SS2 locus tags SSU0573, SSU0592, SSU0593, SSU0594, SSU0595, and SSU0600) and a protein (corresponding to SS2 locus tag SSU0409), respectively, showed 50-93% similarity with those of SS2 (**Table S2**). Taken all together with these findings, the mutants constructed in this study have almost identical genetic background to SS2 compared to the heterogenous genetic background of the different serotype strains.

**Reference (Notes)**

N1. Vinogradov E, Goyette-Desjardins G, Okura M, Takamatsu D, Gottschalk M, Segura M. 2016. Structure determination of *Streptococcus suis* serotype 9 capsular polysaccharide and assignment of functions of the *cps* locus genes involved in its biosynthesis. Carbohydr Res. 433:25-30. doi: 10.1016/j.carres.2016.07.005.

N2. Li C, Duda KA, Elverdal PL, Skovsted IC, Kjeldsen C, Duus JØ. 2019. Structural, biosynthetic, and serological cross-reactive elucidation of capsular polysaccharides from *Streptococcus pneumoniae* serogroup 16. J Bacteriol. 201:e00453. doi: 10.1128/JB.00453-19.

N3. Roy D, Athey TBT, Auger JP, Goyette-Desjardins G, Van Calsteren MR, Takamatsu D, et al. A single amino acid polymorphism in the glycosyltransferase CpsK defines four *Streptococcus suis* serotypes. Sci Rep. 2017;7: 4066. doi: 10.1038/s41598-017-04403-3.

N4. Goyette-Desjardins G, Auger JP, Xu J, Segura M, Gottschalk M. 2014. *Streptococcus suis*, an important pig pathogen and emerging zoonotic agent—an update on the worldwide distribution based on serotyping and sequence typing. Emerg Microbes Infect. 3:e45. doi: 10.1038/emi.2014.45.
